# Assessing the Risk Perception and Knowledge Regarding Cardiovascular Diseases in Patients With Hypertension and Diabetes in Central India: A Mixed-Methods Study

**DOI:** 10.7759/cureus.43935

**Published:** 2023-08-22

**Authors:** Kritika Singhal, Pankaj Prasad, Alka Asati, Veena Melwani

**Affiliations:** 1 Community and Family Medicine, All India Institute of Medical Sciences, Bhopal, Bhopal, IND; 2 Community Medicine, LN Medical College & JK Hospital, Bhopal, IND

**Keywords:** health behavior, cardiovascular risk assessment, risk perception, health belief model, cardiovascular disease

## Abstract

Background

Cardiovascular diseases (CVDs) are a major cause of morbidity and mortality worldwide. The perceptions of patients can be important in health-related behaviors and disease prognosis. Thus, this study aimed to determine the risk perception and knowledge of hypertensive and diabetic patients.

Methodology

The study was conducted among 264 patients with diabetes and/or hypertension. A sequential exploratory mixed-methods design was used, which initially identified themes related to risk perception about CVDs among patients. Knowledge and risk perception about CVDs were quantified using a survey derived from predefined themes. CVD knowledge scores were categorized into low, intermediate, and high scores, and the trends of risk perception were studied across this spectrum of cardiovascular knowledge.

Results

The age of the participants ranged from 30 to 78 years. Overall, 57.19% of the participants were males, whereas 42.80% were females. The qualitative results revealed that the sources of knowledge about CVDs, physical activity, and maintaining a nutritious diet included family, friends, and media. On the other hand, doctors played a significant role in influencing perceptions related to medications. The observation of complications related to CVDs and the fear of mortality played a pivotal role in shaping the perception regarding the seriousness of the condition. Perceived susceptibility was low if there was unawareness of protection through medications and lifestyle changes, but it was high with stress or heredity. The analysis of CVD knowledge and risk perception survey data revealed the median CVD knowledge score to be 12 (interquartile range = 7.5-13), which showed a significant association with education and socioeconomic status (p < 0.05). The distribution of risk perception scores across the spectrum of CVD knowledge scores depicted that participants with higher scores agreed more with CVDs being serious and their susceptibility to them.

Conclusions

Despite having high knowledge scores regarding CVDs, the study population had average risk perception. Patient risk perceptions should be evaluated as it affects the health-seeking behavior and prognosis of the disease.

## Introduction

Non-communicable diseases (NCDs) (cardiovascular diseases (CVDs), cancer, hypertension, diabetes, and chronic respiratory diseases) are influenced by a multitude of physiological, environmental, genetic, and behavioral factors [1]. CVDs and related conditions in India contribute to nearly two-thirds of mortality due to NCDs [2]. Dietary risks contribute the highest to disability-adjusted life years (DALYs) due to CVDs, followed by high systolic blood pressure, tobacco use, high fasting plasma glucose, and high body mass index [3]. To curb the burden of CVDs, diabetes, and hypertension, the Government of India launched a national program called the National Program for Non-Communicable Diseases. The program enlists guidelines about medications and lifestyle modifications to manage NCDs. Apart from population-based screening, these guidelines encourage health promotion through behavior change [4]. At operational levels, clinicians educate patients about the disease, its risk factors, and treatment [5]. The guidelines of the program are generic and lack personalization. They do not factor in patient perceptions which can have an essential role in health-related behaviors and disease prognosis [6]. Further, the standard of procedure for communication of risk, treatment, lifestyle modification, etc., has not been considered. Existing literature points toward inadequate knowledge about CVDs and their risk factors in most communities [7-11]. Any treatment will succeed only if patients have accurate and adequate perceptions, eventually leading to better control of the risk factors and disease [12]. Hence, this study aimed to determine the knowledge and risk perception about CVD among patients diagnosed with hypertension and diabetes mellitus.

## Materials and methods

Study design

A sequential, exploratory, mixed-methods design was used in this study. In-depth interviews were conducted using a semi-structured interview guide based on the constructs of the Health Belief Model, Health Action Process Approach, and Transtheoretical Model of Behavior Change [13-15]. The three models were amalgamated into one interactive model to explore patient perceptions based on themes such as awareness, perceived seriousness about and susceptibility to CVDs, pre-contemplation and contemplation about modifiable behavioral risk factors, perceived benefits and barriers to adoption of favorable behavioral risk factors, preparation for adoption of good behavioral risk factors, self-efficacy, and relapse.

Study setting

This study was conducted in the Screening, Medicine, and Cardiology outpatient departments at All India Institute of Medical Sciences (AIIMS), Bhopal, a tertiary care teaching hospital in central India. The study was conducted from January 2021 to August 2021.

Selection criteria

Participants aged more than 30 years who were residents of Bhopal and were diagnosed with hypertension and/or diabetes and had any one of the four CVD behavioral risk factors (tobacco/alcohol use, physical inactivity, or unhealthy diet) were included in the study. Participants with any known history of coronary artery disease, ischemic heart disease, stroke, congenital heart disease, rheumatic heart disease, or valve disease (verified using existing records or invasive procedures or prescriptions) were excluded.

Sample size and sampling plan

Qualitative interviews were conducted among eligible patients till saturation was achieved. A total of eight interviews were conducted. The sample size for the quantitative tool was calculated by considering the proportion of risk perception of CVDs reported in a study conducted in Nigeria [6]. The final sample size was 263, with a type 1 error of 0.05 and absolute precision of 0.05. Around 10% was taken as non-consent, and the final sample size was calculated as 300. However, the survey was conducted among 264 participants. Convenient sampling was performed wherein the participants visiting the Screening, Medicine, and Cardiology outpatient departments and fulfilling the selection criteria during the study period were included.

Study tools

In-depth interviews were conducted using a semi-structured interview guide. A tool to quantify the knowledge and risk perception of CVDs was constructed from the results of the interviews. It consisted of three sections, namely, sociodemographic details, knowledge, and risk perception. Knowledge was scored out of a total score of 13 where each item was scored 1 for correct responses and 0 for incorrect responses. Risk perception was measured on a Likert scale as “strongly agree,” “agree,” “neutral,” “disagree,” and “strongly disagree” [16]. It was translated into Hindi, followed by expert and peer validation. Subsequently, the questionnaire was pilot tested on 20 individuals not included in the study to detect any ambiguity or technical errors. The tool was uploaded onto KoboCollect for data collection.

Data collection

Participants fitting the inclusion criteria were approached in the General Medicine, Screening, and Cardiology outpatient departments at AIIMS, Bhopal. Informed written consent was taken before interviews which lasted for an hour each. Audio records were transcribed in Microsoft Word and translated into English. Regarding the knowledge and risk perception tool, an online link was shared via WhatsApp or administered by two data collectors. It took approximately 20 minutes for each patient to fill out the tool.

Data analysis

The translated interviews were uploaded onto RQDA version 0.3-1 (R for Qualitative Data Analysis) for coding. Thematic analysis was performed, and a deductive approach was used to identify further subthemes and codes. The model constructs could describe most of our data. Quantitative data analysis was done using R version 4.1.2. Descriptive statistics were reported as frequency and percentage. Association studies were performed using the chi-square and Fisher’s exact test, with a p-value <0.05 considered statistically significant. The knowledge scores and risk perception responses were expressed as median with interquartile range. An independent t-test was used for comparing the scores among the categorical variables.

## Results

Knowledge and risk perception survey

The knowledge and risk perception survey was initiated by approaching 300 patients, of whom 264 were eligible to participate in the survey. Table 1 depicts the distribution of participants’ sociodemographic characteristics.

**Table 1:** Distribution of study participants according to sociodemographic characteristics. *: per the Modified Kuppuswamy Scale 2021 [17].

Participant characteristics	Frequency (%) (n = 264)
Gender	
Male	151 (57.19%)
Female	113 (42.80%)
Age (in years)	
30–49	93 (35.23%)
50–69	149 (56.44%)
69 and above	22 (8.33%)
Education	
Below high school	174 (65.91%)
High school and above	90 (34.09%)
Occupation	
Employed	147 (55.68%)
Unemployed	117 (44.31%)
Socioeconomic status*	
Upper I	13 (4.92%)
Upper Middle II	45 (17.04%)
Lower Middle III	57 (21.59%)
Upper Lower IV	113 (42.80%)
Lower V	36 (13.64%)

The age of the participants ranged from 30 to 78 years (mean = 52.5, SD = 10.69), where 151 (57.2%) were males and 113 (42.8%) were females. All patients had hypertension and/or diabetes. The disease duration ranged from six months to 40 years (mean = 6.64, SD = 7.34). Of the total participants, 213 (80.7%) had heard about heart diseases, while 51 (19.3%) had never heard about heart diseases. Further, 89.2% (83) of all participants in the youngest age group (30-49 years) had heard about heart diseases compared to the proportions in older age groups of 50-69 years and 69 years and above (p = 0.010). Gender was also significantly associated with having heard about heart diseases (p ≤ 0.001), with more males (88.1%) having heard about it than females (70.8%). Table 2 illustrates the relationship between hearing about heart diseases and sociodemographic characteristics. Education, occupation, and socioeconomic status were significantly associated with having heard about heart diseases (p < 0.05).

**Table 2:** Relationship between having heard about heart diseases and sociodemographic characteristics. *: per the Modified Kuppuswamy Scale (2021) [17]; #: number of years since the participants were diagnosed with hypertension/diabetes irrespective of whether they are on treatment.

Have you heard about heart disease?	Yes (%) (n = 213)	No (%) (n = 51)	P-value
Age (in years)			
30–49	83 (89.25%)	10 (10.75%)	0.010
50–69	116 (77.85%)	33 (22.15%)	
69 and above	14 (63.64%)	8 (36.36%)	
Gender			
Male	133 (88.08%)	18 (11.92%)	0.000
Female	80 (70.79%)	33 (29.20%)	
Education			
Below high school	43 (47.77%)	47 (52.22%)	0.000
High school and above	170 (97.70%)	4 (2.30%)	
Occupation			
Employed	125 (85.03%)	22 (14.97%)	0.045
Unemployed	88 (75.21%)	29 (24.79%)	
Socioeconomic status*			
Below lower middle III	100 (67.11%)	49 (32.89%)	0.000
Lower middle III and above	113 (98.26%)	2 (1.74%)	
Duration of disease (hypertension/diabetes) (in years)#			
6 years and below	142 (81.14%)	33 (18.86%)	0.790
7 years and above	71 (79.78%)	18 (20.22%)	

Disease duration was not significantly associated with having heard about heart diseases. Responses to the 13 items related to the knowledge about heart diseases are shown in Table 3.

**Table 3:** Responses to cardiovascular knowledge items. Maximum responses were correct for all items except “If parents have a heart disease, their children are at a higher risk of developing a heart disease in the future” which had more incorrect responses.

Item	N = 213	
	Correct response	Incorrect response
If I am consuming an unhealthy diet, I will get heart disease	164 (77%)	49 (23%)
If am not doing any physical activity, I can get heart disease	158 (74.2%)	55 (25.8%)
Obesity can cause heart diseases	162 (76.1%)	51 (23.9%)
Smoking can cause heart diseases	170 (79.8%)	43 (20.2%)
Alcohol can cause heart diseases	176 (82.6%)	37 (17.4%)
High blood pressure can cause heart diseases	172 (80.8%)	41 (19.2%)
High blood sugar/diabetes can cause heart diseases	138 (64.8%)	75 (35.2%)
If I miss my blood pressure medicines, I can get heart disease	167 (78.4%)	46 (21.6%)
If I miss my diabetes medicines, I can get heart disease	132 (62%)	81 (38%)
Blood thickening causes heart diseases	189 (88.7%)	24 (11.3%)
Cholesterol causes heart diseases	154 (72.3%)	59 (27.7%)
Stress causes heart diseases	195 (91.5%)	18 (8.5%)
If parents have heart disease, their children are at a higher risk of developing heart disease in the future	89 (41.8%)	124 (58.2%)

**Figure 1:**
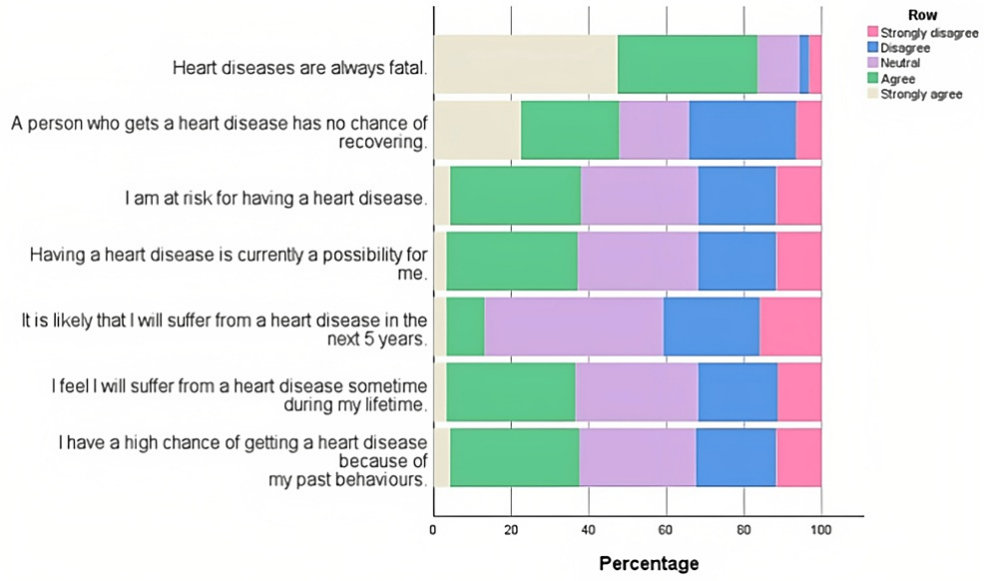
Distribution of responses to the items of risk perception about cardiovascular diseases. A horizontal stacked bar chart depicting the responses to risk perception about cardiovascular diseases shows that the maximum responses oscillated around agree to disagree. The item about heart disease being always fatal showed strongly agree as the most popular response.

Items related to unhealthy diet, physical inactivity, and obesity causing heart diseases were answered correctly by 164 (77%), 158 (74.2%), and 162 (76.1%) participants, respectively. The items about smoking and alcohol causing heart diseases received 170 (79.8%) and 176 (82.6%) correct responses, respectively. Further items related to hypertension and diabetes causing heart diseases were answered correctly by 172 (80.8%) and 138 (64.8%) participants, respectively. Items on heart diseases due to missing antihypertensive and diabetic medications elicited correct answers from 167 (78.4%) and 132 (62%) participants, respectively. Each item was scored 1 for correct responses and 0 for incorrect responses, with a total score of 13. The median score was 12 (IQR = 7.5-13). Participants with higher levels of education (high school and above) demonstrated a significantly higher score (t = -8.1, p ≤ 0.001) about knowledge of heart diseases than participants with a lower level of education (below high school). In addition, higher socioeconomic status (lower middle third and above) displayed a significantly higher score (t = -3.9, p ≤ 0.001) compared to lower socioeconomic status (below lower middle third). There was no significant difference in scores across genders, age groups, employment, and disease duration. Risk perception about CVDs was assessed using the five-point Likert scale. Figure 1 illustrates the risk perception item-wise distribution of the responses.

**Figure 2:**
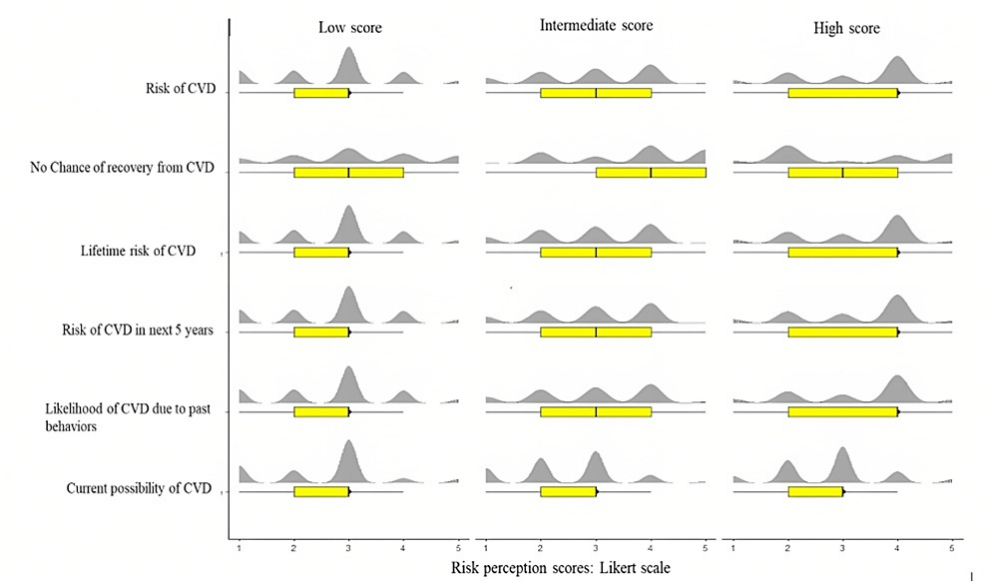
Distribution of risk perception scores across the spectrum of cardiovascular disease knowledge score. A horizontal boxplot plotted with responses of risk perception across the tertiles of cardiovascular disease knowledge score depicts that participants with high scores agreed more to the risk perception items except for the items about their current possibility of heart disease and that there is no chance of recovery from heart diseases where they mostly disagreed and gave neutral responses, respectively.

The majority of participants strongly agreed that heart diseases are always fatal. An almost equal proportion of participants strongly agreed, agreed, and disagreed with heart disease having no chance of recovery. When asked about participants’ risk of developing heart disease, they either agreed or mostly gave neutral responses. The responses were similar for the current and lifetime possibility of developing heart disease. While for the chance of developing heart disease in the next five years, participants mostly disagreed. When asked about the likelihood of getting a heart disease due to past behaviors, participants mostly agreed or gave a neutral response. A horizontal boxplot was constructed to study the trends of risk perception across the CVD knowledge scores (Figure 2).

The knowledge scores were categorized according to tertiles into low, intermediate, and high scores. Participants with high scores agreed more with the risk perception items except for the items about their current possibility of heart disease and that there is no chance of recovery from heart diseases, where they mostly disagreed and gave neutral responses, respectively. Those with intermediate and low scores reported neutral responses. Participants with high scores mostly disagreed on the item concerned with no chance of recovery from heart disease. In contrast, those with intermediate and lower scores gave neutral responses or agreed with it.

In-depth interviews

Eight patients with hypertension and/or diabetes were interviewed to understand patient perceptions about diseases of concern, treatment advised by the physician, and understanding of modifiable risk factors as well as factors that facilitate or prevent them from following adequate behaviors. The results are presented in the subsections of Disease Perception and Modifiable Behavioral Risk Factors. Table 4 depicts the themes, subthemes, and verbatims.

**Table 4:** Summary of qualitative results. Under the domains of disease perception and modifiable behavioral risk factors, various themes and subthemes with verbatims have been summarized.

Themes	Subthemes	Inference	Verbatim
Awareness	CVDs, physical activity, healthy diet	Sources of awareness are family/friends and media	“…heard it from people…read articles, papers…husband had a minor attack once.” “…read in madhurima that walk up and down the stairs and do sweeping…”
	medication	Source are doctors	
Perceived seriousness	CVDs	Witnessed complications and fear of death influence perceived seriousness	“…attack comes, you don’t get time to survive….” “…can get a heart attack; a person might die. Paralysis…is even a bigger problem”
Perceived susceptibility	CVDs	Low if there is unawareness of protection through medication	“….he should not get the disease…I keep taking medicines. I do exercise, I walk”
		High with stress or heredity	“…mine is hereditary…”
Contemplation stage	Desirable behaviors: medication adherence, physical activity, and a healthy diet	Aware of the desirability of these behaviors	“heard that the medicine for someone who has sugar never stops”
Maintenance stage		Desirable behaviors sustained by physician advice and family support	“…doctor said, after taking breakfast, you have to take medicine.” “In the evening I make time for walking.” “My husband supports me…instead of spending on diseases we should spend on food…eat healthy”
Perceived barriers		Barriers to adoption/maintenance of desirable behaviors are lack of interest/time, unavailability, and side effects	“…if I remember I’ll eat it….” “…I don’t get time for exercise.” “…medicines it will affect my kidney…”
Pre-contemplation	Undesirable behaviors: tobacco/high salt/inadequate fruits and vegetables	No awareness of behavior change	“I don’t think too much.” “Salt and sugar should be appropriate…otherwise…not palatable.” “I eat what is cooked at home”
Relapse		Failure to maintain changed behaviors	“…friends, so they feed tobacco to me forcefully…”
Perceived barriers		Barriers to change of undesirable behaviors are peer pressure or misinformation	“…if I meet my 4 to 5 friends…even one grain of tobacco I get the taste…”

Disease Perception

Awareness regarding CVDs was due to heart/paralytic attacks in family or friends, television, and newspapers. Awareness about medication was from treating physicians. At the same time, awareness about physical activity and healthy diet was from family members or neighbors. Perceived seriousness about heart diseases was from family, friends, and treating physicians. The fear of mortality contributed to the perceived seriousness. Patients on regular medication did not feel susceptible to heart disease. Instead, they reported that they may develop it because it is hereditary.

Modifiable Behavioral Risk Factors

Medication adherence: Patients with prescribed medications for hypertension or diabetes had good adherence and were in the maintenance phase. Maintenance self-efficacy was due to the habit of regular medication consumption and the perceived benefit of blood pressure or glucose control. Medications placed at sites such as “near the bed” or “in a see-through bag hung on the door” also helped. Further, family members provided reminders about medication which helped with maintenance. Perceived barriers included side effects and chronicity of disease and treatment.

Tobacco usage: Tobacco users were well aware of the effects of tobacco usage. They were in the contemplation phase of behavior change regarding quitting tobacco. Multiple incidents of relapse led them back to contemplation. The main perceived barrier to stopping tobacco was peer pressure. Other barriers included the perception of relief from stress on consuming tobacco. Patients not consuming any tobacco were well aware of the harm. They had no intention of starting its use anytime in the future.

Alcohol use: Patients were well aware of the harmful effects of alcohol. They had no intention of starting its use anytime in the future.

Diet: Patients with good medication adherence reported low salt, sugar, and oil intake. The maintenance stage was due to the perceived benefits of such a diet, as advised by treating physicians. Moreover, blood pressure, glucose control, and prevention of “a paralytic attack” reinforced this diet further. The diagnosis of hypertension/diabetes was a cue to start a low-salt, sugar, and oil diet. Patients who had been consuming this diet since very early in life gave credit to the regular availability of such food. Perceived barriers to a healthy diet were the household habit of regularly consuming high amounts of salt, sugar, and oil. The taste of food also warranted the consumption of such a diet. Fruits and vegetables were considered to be a part of a healthy lifestyle. Those in the maintenance phase were motivated to consume it due to childhood habits and support from family. Consumption of“fruit once in a while,” “high cost,” and “monotonous taste” were perceived barriers.

Physical activity: Patients were in the maintenance phase of regular physical activity as they started doing it to lose weight and stay healthy even before they were diagnosed. They perceived it to be a part of a healthy lifestyle and an added benefit of prevention from developing other diseases related to weight. Another benefit reported was that regular physical activity would keep blood pressure in control. The barriers to continuing physical activity were the lack of interest, the inability to find time for physical activity, and the COVID-19 pandemic due to restrictions on movement in public places. Barriers to the maintenance of regular physical activity included breathlessness, chest pain, and diseases of joints or nerves, among others.

## Discussion

The objective of this study was to assess the knowledge and risk perception about CVDs. The process of behavior change starts with the shaping of risk perception. As reported in this study, awareness and knowledge about CVDs and risk factors can be traced back to family, friends, and social media. Social media includes newspapers, television, and online social media platforms. Some communities in Malawi believe that witchcraft was the reason for stroke and were unaware of the actual risk factors [18].

Various studies have assessed the knowledge about CVDs. A mixed-methods study from Nigeria reported low-to-average knowledge [7]. It was even absent in some cases of type 2 diabetes [8]. A lack of ability to connect risk factors to CVDs, hypertension, and diabetes was also observed in another study conducted among urban residents [10]. This could be because of misinformation received from the sources reported. Thus, the knowledge about CVDs varies considerably. The scores were categorized as low, intermediate, and high for studying the trends across risk perception. Verma et al. assessed CVD knowledge of patients with metabolic diseases and divided their scores similarly into poor, average, and good. Most participants responded correctly to all items of the CVD knowledge score. Further, in our study, the scores were significantly affected by education and socioeconomic status, i.e., participants with higher education and socioeconomic status had higher knowledge scores. Similar findings were reported in a study conducted among patients with metabolic syndrome in India [11].

Knowledge gives rise to risk perception, which has two components, namely, perceived seriousness and perceived susceptibility. Perceived seriousness was reported to be influenced by witnessing adverse events such as stroke, myocardial infarction, and paraplegia in acquaintances. Fear of mortality also had the same source, inspiring perceived seriousness. In contrast, susceptibility to heart diseases was perceived to increase with stress. Genetic predisposition also increases the perception of vulnerability to them. On the other hand, protection from CVDs was perceived in the presence of medication adherence. Perceived seriousness and susceptibility to CVDs were reported as low to average across various studies [8,18,19]. Another study reported that perceived susceptibility to heart disease was related to destiny [8]. A study conducted in Nigeria reported that protection from heart disease was perceived due to adherence to regular physical activity and lack of family history of heart disease [7].

Most participants’ responses to risk perception items oscillated between “agree” and “neutral,” but most strongly agreed that heart diseases are always fatal. Participants with higher knowledge scores had a higher agreement about CVDs being serious and their susceptibility to them. Those with lower and intermediate scores had neutral opinions about seriousness and vulnerability. Although our findings suggest a relationship between CVD knowledge and risk perception, other studies had differing findings [7].

Thus, adequate knowledge charts the course for developing appropriate risk perception. Once a perception is molded, expectations evolve, increasing the understanding of the disease. Participants reported that they had no awareness about or intention to change undesirable behaviors, while they had an awareness about desirable behavior change. Hence, concerning the former, they were in the pre-contemplation stage, while concerning the latter, they were in the contemplation phase. Our findings show that the Health Belief Model, Health Action Planning Approach Model, and Transtheoretical Model of Behavior Change help recognize the determinants of risk perception and the behavior change process. These models have been used elsewhere to form the foundation of counseling and health education to enable medication adherence, regular physical activity, and a healthy diet [14,20,21].

This study had a few limitations. As this was a hospital-based study, generalizability can be enhanced by conducting a similar project in community settings. Further, the severity of hypertension and diabetes was not assessed which might have a significant bearing on the knowledge and perception. Lastly, due to a limited study period, convenient sampling had to be performed to complete the sample size.

## Conclusions

CVD knowledge and risk perception were explored in patients with hypertension and diabetes who had any one of the behavioral risk factors. The results indicated that the majority of participants had high cardiovascular knowledge and average risk perception. Every patient diagnosed with hypertension and/or diabetes should go through risk assessment and communication to improve perception and knowledge about CVDs. Adequate perception and knowledge will help better comprehend the diagnosis and management and chart an appropriate disease prognosis trajectory. It is recommended to utilize the one-minute preceptor model of teaching during consultation. In the context of this study, it will involve articulation of the diagnosis by the patient followed by an assessment of their knowledge by the clinician. A summary of the entire communication can be the take-home message.
